# Sniffer cells for the detection of neural Angiotensin II *in vitro*

**DOI:** 10.1038/s41598-019-45262-4

**Published:** 2019-06-19

**Authors:** George E. Farmer, Anna Amune, Martha E. Bachelor, Phong Duong, Joseph P. Yuan, J. Thomas Cunningham

**Affiliations:** 1 Department of Physiology and Anatomy, University of North Texas Health Science Center at Fort Worth, Fort Worth, TX 76107 United States; 2 Texas A&M University, College Station, TX United States

**Keywords:** Neurotransmitters, Neurophysiology

## Abstract

Neuropeptide release in the brain has traditionally been difficult to observe. Existing methods lack temporal and spatial resolution that is consistent with the function and size of neurons. We use cultured “sniffer cells” to improve the temporal and spatial resolution of observing neuropeptide release. Sniffer cells were created by stably transfecting Chinese Hamster Ovary (CHO) cells with plasmids encoding the rat angiotensin type 1a receptor and a genetically encoded Ca2+ sensor. Isolated, cultured sniffer cells showed dose-dependent increases in fluorescence in response to exogenously applied angiotensin II and III, but not other common neurotransmitters. Sniffer cells placed on the median preoptic nucleus (a presumptive site of angiotensin release) displayed spontaneous activity and evoked responses to either electrical or optogenetic stimulation of the subfornical organ. Stable sniffer cell lines could be a viable method for detecting neuropeptide release *in vitro*, while still being able to distinguish differences in neuropeptide concentration.

## Introduction

The renin-angiotensin system (RAS) is important in the regulation of body fluid homeostasis and blood pressure. Angiotensin II (ANG II), a product of the RAS, has effects on numerous organ systems including arterioles, adrenal gland, kidneys, and brain. The RAS and the synthesis of ANG II is generally considered to be a systemic system; however, there are reports of local or tissue specific RAS’s^1,2^. Relevant to the current study, evidence suggests that ANG II may be synthesized in and used as a neurotransmitter within the brain^2,3^. Angiotensin II receptors are abundant in the brain^4^ and have been shown to effect the excitability of neurons *in vitro*^5–7^. Further evidence in favor of a brain RAS include the detection of all biosynthetic components required for synthesis of ANG II within the brain^8^. However, there is still debate as to whether the source of ANG II is from the periphery^9^ or from local synthesis.

Angiotensin II signaling in the brain is of particular interest because of its effects on blood pressure and its contribution to hypertension^10–13^. The brain is sensitive to circulating ANG II through circumventricular organs, such as the subfornical organ (SFO) and the organum vaculosum of the lamina terminalis (OVLT). Activation of ANG II receptors in the SFO and OVLT can influence blood pressure and thirst^14–17^. The median preoptic nucleus (MnPO) and the paraventricular nucleus (PVN) are also sensitive to ANG II and are involved in the regulation of body fluid homeostasis and blood pressure^18–21^. However, both the MnPO and PVN lie within the blood-brain barrier and are, therefore, inaccessible to circulating ANG II. Angiotensin II signaling within the brain is poorly understood, but study of release dynamics can elucidate the contribution of ANG II to the genesis and maintenance of some forms of hypertension. Recently, the existence of a brain RAS and, specifically, whether or not ANG II is generated in the CNS has been questioned^9,22^. Measuring the release of ANG II and related peptides in the central nervous system would be an important step to determining if there is a brain RAS and what role it plays in pathophysiology.

Measuring the release of neuropeptides has traditionally been difficult to accomplish. Microdialysis is not always conducive to large molecules, exhibits poor temporal and spatial resolution, and may exhibit poor sensitivity. High-pressure liquid chromatography is a more sensitive method for the detection of neuropeptides but is expensive and still subject to the same impairments in temporal and spatial resolution as microdialysis. Genetically modified HEK or CHO cells have recently been used to measure vasopressin^23,24^ or oxytocin^25,26^ release in brain slices. This approach has also been combined with patch-clamping to study GABA and glutamate release^27,28^ and optogenetics to study peptide release from axons transfected with channel rhodopsin^25,26^.

To answer questions related to the release of ANG II within neural tissue, we have adopted this new approach to study the brain renin angiotensin system. Using Chinese Hamster Ovary (CHO) engineered to stably express both an angiotensin receptor and a genetically encoded Ca^2+^-sensor, we demonstrate the use of these “sniffer cells” as an effective method for the detection of ANG II release in the SFO-MnPO pathway *in vitro*.

## Results

**Figure 1:**
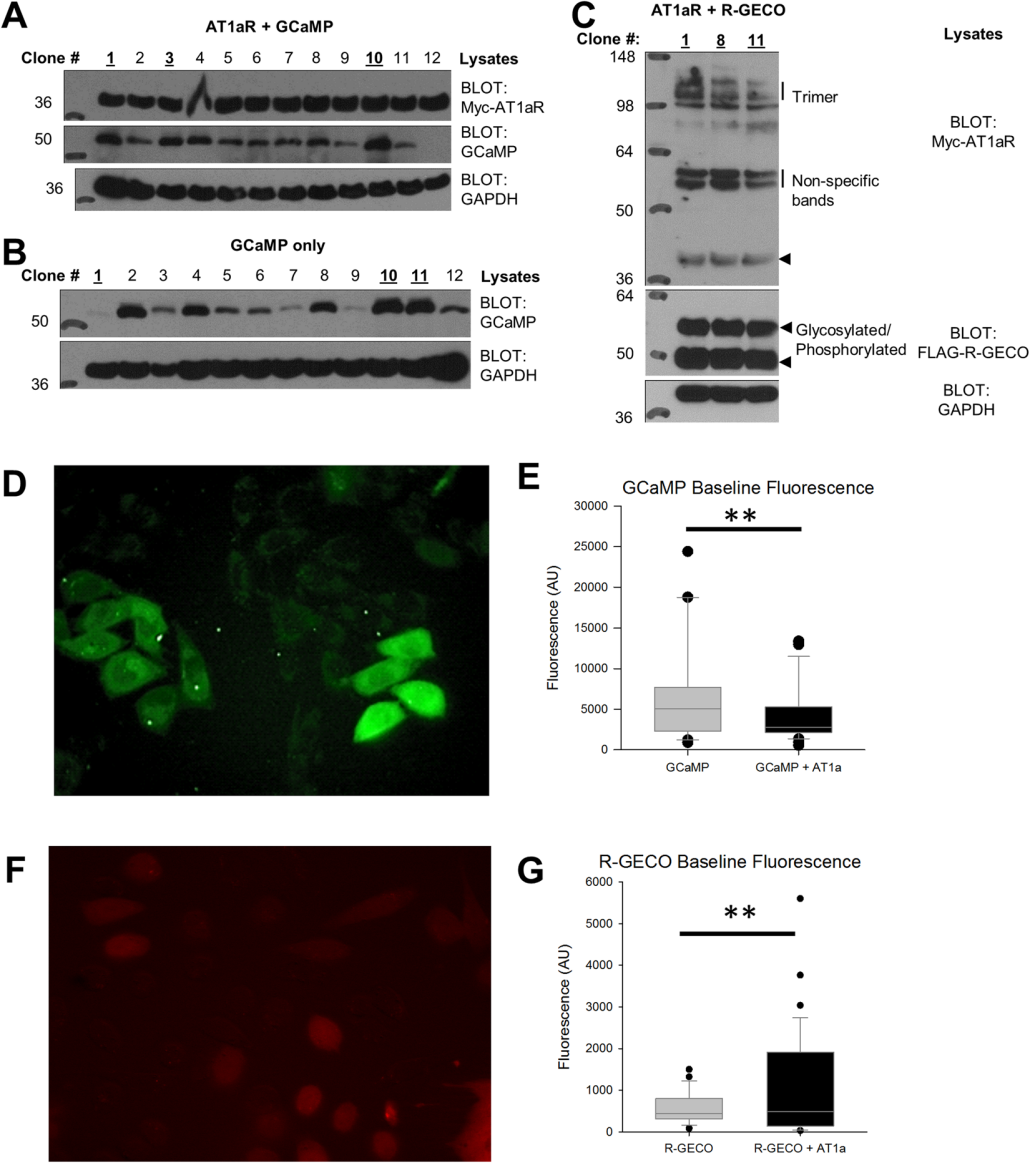
Cropped Western blots showing selection of sniffer cells - stably transfected CHO cell colonies expressing high levels of (**A**) GCaMP + AT1aR, (**B**) GCaMP only, and (**C**) R-GECO + AT1aR (see Supplemental Information for full Western blots). Optimally expressed colonies (bolded and underlined) selected: 1, 3, and 10 for GCaMP + AT1aR; 1, 10, and 11 for GCaMP only; and 8 and 11 for R-GECO + AT1aR. Sniffer cells exhibit variable baseline fluorescence. (**D**) GCaMP + AT1aR sniffer cells plated on coverslips exhibit variable levels of GCaMP fluorescence. (**E**) The variability in sniffer cell fluorescence is not different between CHO cells transfected with GCaMP or GCaMP + AT1aR. (**F**) Sniffer cells plated on coverslips exhibit variable levels of R-GECO fluorescence. (**G**) The variability in sniffer cell fluorescence is not different between CHO cells transfected with R-GECO or R-GECO + AT1aR. F-Test **p < 0.01.

Figure 1A–C show cropped Western blots (see Supplementary Information for full Western blots images) illustrating that CHO cells were stably transfected with GCaMP or R-GECO plasmids and expressed their corresponding proteins. Further analysis revealed that the c-myc antibodies used to infer AT1aR expression in the CHO cells were non-specific and were detected in wild type CHO cells as well as CHO cells transfected with only the GCaMP or R-GECO plasmid (shown in Supplemental Figs 2, 3, n = 4). Additionally, different c-myc antibodies yielded different band profiles in the western blots. Therefore, colonies with high expression of GCaMP or R-GECO and apparent expression of AT1aR (maximum of 3) were selected for further functional analysis, and these colonies became our sniffer cells (Fig. 1A–C Colonies 1, 3, and 10 for GCaMP + AT1aR; colonies 1, 10, and 11 for GCaMP only; and colonies 8 and 11 for R-GECO + AT1aR).

Individual GCaMP + AT1aR sniffer cells exhibited variability in baseline fluorescence (F-Test p < 0.01, Fig. 1D,E). However, there were no significant differences in baseline fluorescence in CHO cells transfected with GCaMP (n = 29) or GCaMP + AT1aR (n = 38, p = 0.12). Individual R-GECO + AT1aR sniffer cells also exhibit a variability in baseline fluorescence (F-Test p < 0.01, Fig. 1F,G).

**Figure 2:**
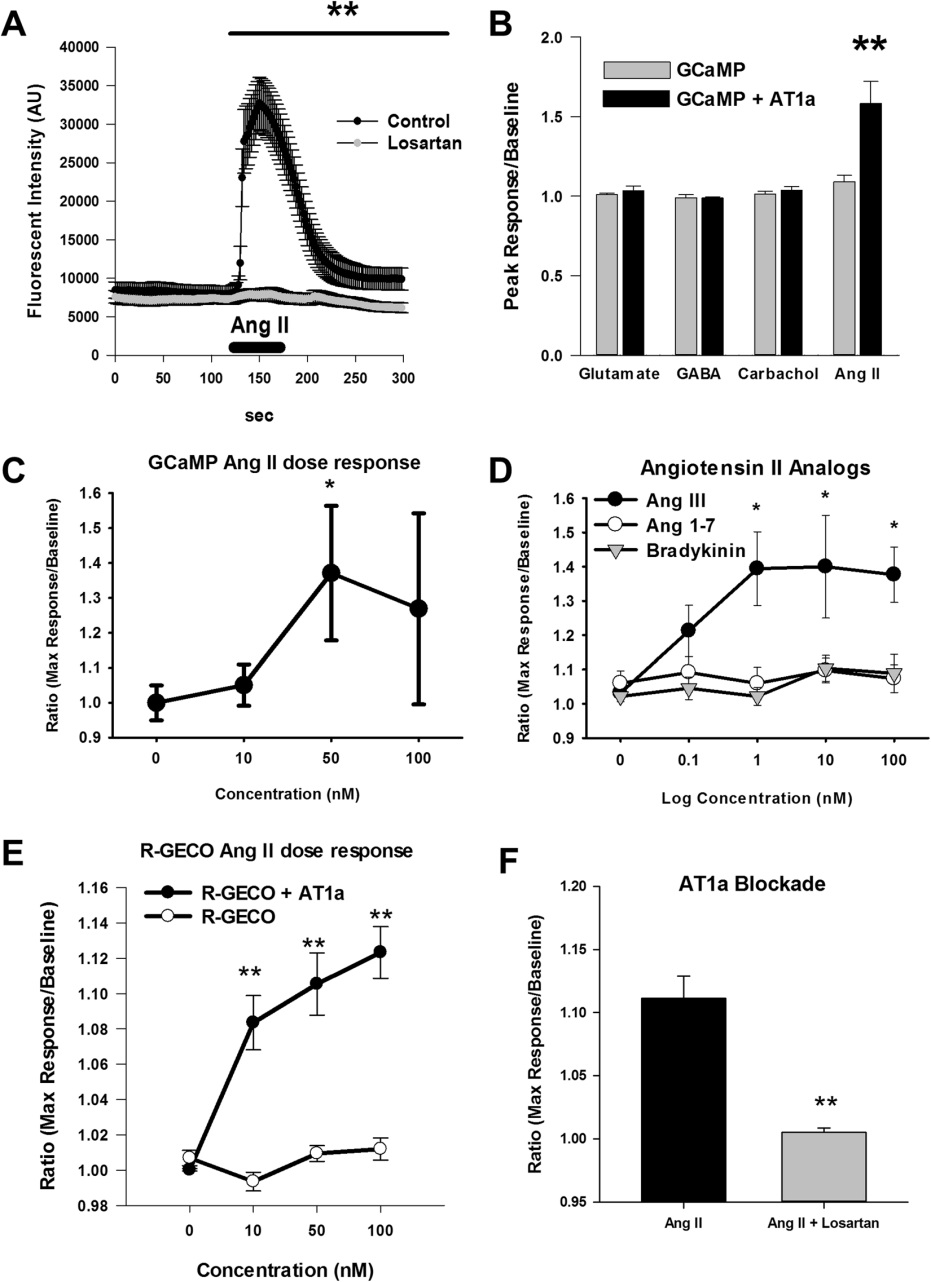
ANG II mediated increases in sniffer cell fluorescence. (**A**) ANG II (100 nM) induced a robust but transient increase in GCaMP fluorescence that was blocked by the AT1aR receptor antagonist Losartan (10 µM). Control n = 39, Losartan n = 38. (**B**) Data shows that bath application of glutamate (50 µM), GABA (50 µM), and carbachol (50 µM) failed to change fluorescent intensity of sniffer cells transfected with GCaMP (n = 29) or GCaMP + AT1aR (n = 38). ANG II (100 nM) did increase fluorescent intensity of sniffer cells, but only in sniffer cells transfected with GCaMP + AT1aR. Dose-dependent effects of ANG II and related compounds were also measured. (**C**) GCaMP + AT1aR sniffer cells exhibit dose-dependent increases in fluorescence in response to bath application of ANG II (100 nM, n = 10). (**D**) Bath application of ANG III induced a dose-dependent increase in GCaMP + AT1aR sniffer cell fluorescence. Bath application of ANG (1–7) or bradykinin did not induce a change in GCaMP + AT1aR sniffer cell fluorescence at any of the doses tested (0.1–100 nM, n = 17–41). (**E**) R-GECO + AT1aR sniffer cells exhibit dose-dependent increases in fluorescence in response to bath application of ANG II (n = 17). R-GECO only cells did not respond to ANG II (n = 27). (**F**) ANG II-mediated increases in R-GECO + AT1aR are blocked by bath application of Losartan (10 µM, n = 17). *p < 0.05, **p < 0.01.

Bath application of 100 nM angiotensin II produced a rapid increase in GCaMP fluorescence that peaked ~370% above baseline 30 sec following the start of the ANG II application (Fig. 2A). However, ANG II failed to produce a change in GCaMP fluorescence in the presence of 10 µM Losartan. Some sniffer cells showed spontaneous increases in fluorescence (shown in Supplemental Fig. 4). In these sniffer cells irregular fluctuations in fluorescence were routinely observed and these cells did not respond to ANG II. Additionally, the irregular fluctuations were not affected by bath application of Losartan. Sniffer cells with these spontaneous responses were considered unhealthy and not included in further analyses. A two-way repeated measure ANOVA showed a significant interaction between time and losartan treatment (F(149,11026) = 22.27, p < 0.001). ANG II produced a transient increase in GCaMP fluorescence in sniffer cells (p < 0.01) which was blocked by Losartan treatment (p < 0.01).

To further characterize responses, sniffer cells transfected with GCaMP or GCaMP + AT1aR were exposed to 1 min bath applications of either glutamate, GABA, carbachol, or ANG II (Fig. 2B). Two-way ANOVA showed there was a significant effect of gene transfection (F(1,296) = 6.87, p < 0.01) and drug application (F(2,296) = 27.73, p < 0.001). Sniffer cells that were transfected with only GCaMP did not show a significant change in fluorescence in response to glutamate, GABA, carbachol, or ANG II application. Sniffer cells transfected with GCaMP + AT1aR were similarly insensitive to glutamate, GABA and carbachol. However, GCaMP + AT1aR sniffer cells showed an increase in fluorescence in response to ANG II exposure compared to GCaMP only sniffer cells (p < 0.01). There was no difference in responses between GCaMP and GCaMP + AT1aR sniffer cells when treated with glutamate (p = 0.45), GABA (p = 0.61), or carbachol (p = 0.39).

**Figure 3:**
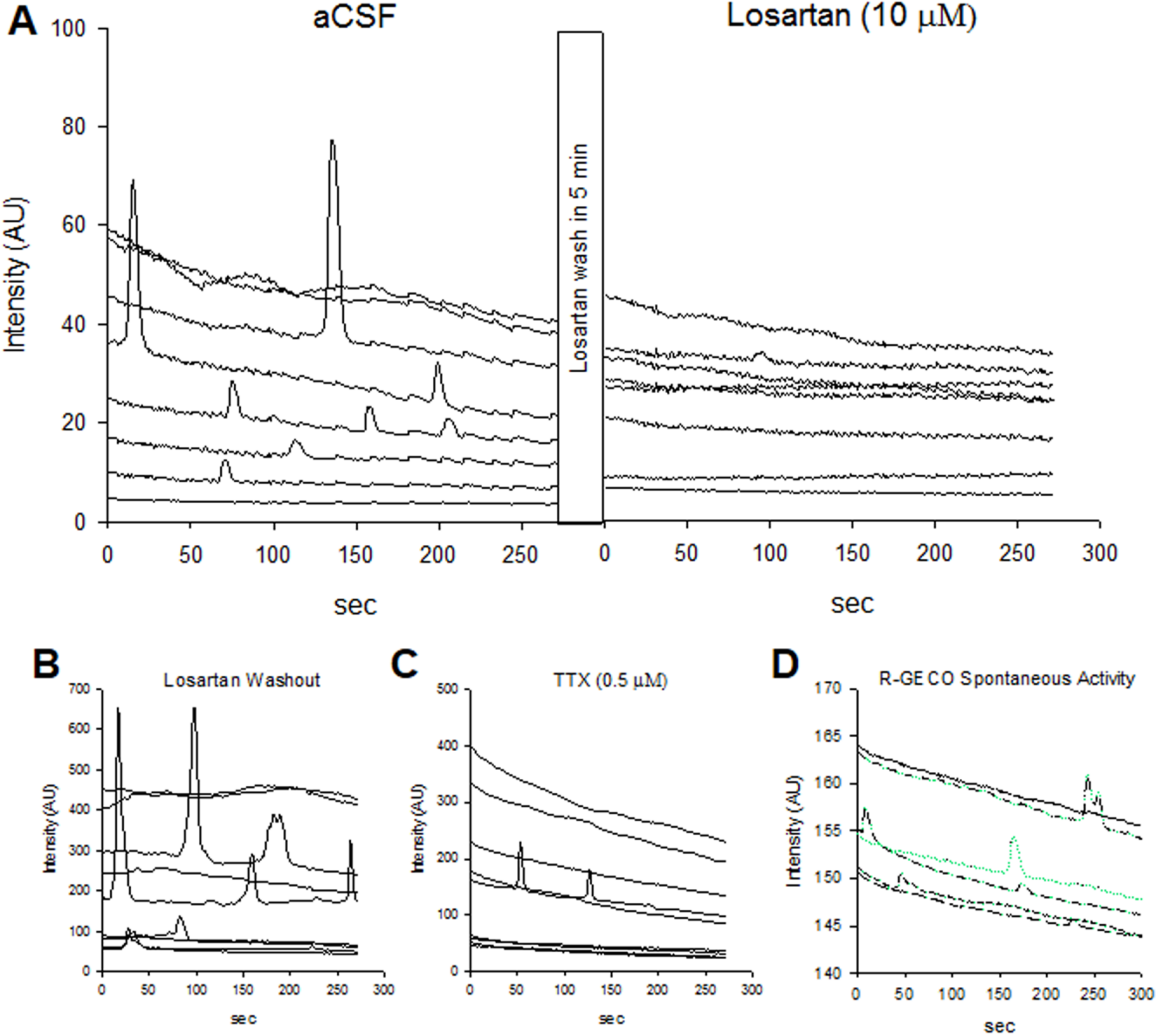
Sniffer cells exhibit spontaneous increases in fluorescence on *in vitro* brain slices. (**A**) GCaMP + AT1aR sniffer cells placed on the MnPO exhibit spontaneous increases in fluorescence that are completely blocked by bath application of Losartan (10 µM). (**B**) Spontaneous sniffer cell responses return following washout of bath applied Losartan. (**C**) Bath application of TTX (1 µM) was also effective in reducing the number of spontaneous sniffer cell responses. (**D**) R-GECO + AT1aR sniffer cells also exhibit spontaneous increases in fluorescence on *in vitro* brain slices. Each trace represents an individual sniffer cell.

The GCaMP + AT1aR sniffer cells (n = 10) responded to ANG II doses as little as 10 nM and reached maximal responses with doses of 50 nM (Fig. 2C). ANG II produced a significant dose-dependent increase in sniffer cell fluorescence (F(3,36) = 3.36, p < 0.05). Two-way ANOVA of sniffer cells treated with ANG III, ANG (1–7), or bradykinin (Fig. 2D) showed a significant effect of treatment (F(2,371) = 5.39, p < 0.01) and dose (F(4,371) = 3.30, p < 0.01). There was a left shift in the dose response curve for sniffer cells exposed to ANG III compared to ANG II with ANG III eliciting a maximal response at 10 nM. Sniffer cells were sensitive to ANG III and showed a significant increase in fluorescence in response to 1 nM (n = 17, p < 0.05), 10 nM (n = 23, p < 0.05), and 100 nM (n = 39, p < 0.05) when compared to control (n = 13; Fig. 3C). Sniffer cells were able to detect 0.1 nM ANG III (n = 41), although the response was not significantly different from control (p = 0.36). Angiotensin (1–7) (0.1 nM n = 41, 1 nM n = 17, 10 nM n = 23, 100 nM n = 35) or bradykinin (0.1 nM n = 36, 1 nM n = 17, 10 nM n = 23, 100 nM n = 35) did not significantly influence sniffer cell fluorescence with any of the dose that were tested when compared to control (n = 13, Fig. 2D).

The R-GECO + AT1aR sniffer cells (n = 17) responded to ANG II doses as little as 10 nM and reached maximal responses with doses of 50 nM whereas cells transfected with R-GECO only (n = 27) did not respond to ANG II (Fig. 2E). No spontaneous activity was observed in the R-GECO + AT1aR sniffer cells. ANG II produced a significant dose dependent increase in R-GECO + AT1aR sniffer cell fluorescence (F(3,64) = 16.83, p < 0.01). However, ANG II failed to produce a change in R-GECO fluorescence in the presence of 10 µM Losartan (Fig. 2F Mann-Whitney rank sum test, p < 0.01, n = 17). Cells transfected with R-GECO only did not exhibit dose dependent increases in fluorescence in response to ANG II (F(3,107) = 2.57, p = 0.06).

GCaMP + AT1aR sniffer cells were placed on 14 slices from 8 rats and we observed spontaneous increases in fluorescence in cells placed on the MnPO (Fig. 3A). In sniffer cells placed on the MnPO, 26% (73 of 274 cells from 14 slices in 8 rats) of the sniffer cells exhibited spontaneous increases in fluorescence. There was an average of 2.4 spontaneous events per 5 min recording (0.008 Hz) in sniffer cells that exhibit spontaneous responses. The spontaneous fluorescent responses peaked at 64.4 ± 7.1% above baseline fluorescence. Bath application of Losartan (10 µM) completely blocked (13 of 13) the spontaneous increases in sniffer cell fluorescence (Fig. 3A). Upon washout of Losartan, 25% of sniffer cells (2 of 8) showed spontaneous increases in fluorescence (Fig. 3B). The spontaneous sniffer cell responses observed in the brain slice preparation were not irregular (as was sometimes observed in the cell culture experiments above) and were blocked by Losartan. This indicates the spontaneous responses in the brain slice preparation were mediated by activation of AT1aRs on healthy sniffer cells. The spontaneous increases in fluorescence were reduced to 10% of the sniffer cells (1 of 10) in the presence of TTX (1 µM, Fig. 3C). R-GECO + AT1a sniffer cells were placed on 2 slices from 1 rat and we observed spontaneous increases in fluorescence in cells placed on the MnPO (Fig. 3D). In sniffer cells placed on the MnPO, 54% (24 of 44 cells from 2 slices in 1 rat) of the sniffer cells exhibited spontaneous increases in fluorescence. There was an average of 2.2 spontaneous events per 5 min recording (0.007 Hz) in sniffer cells that exhibit spontaneous responses. The spontaneous fluorescent responses peaked at 2.2 ± 0.1% above baseline fluorescence.

**Figure 4:**
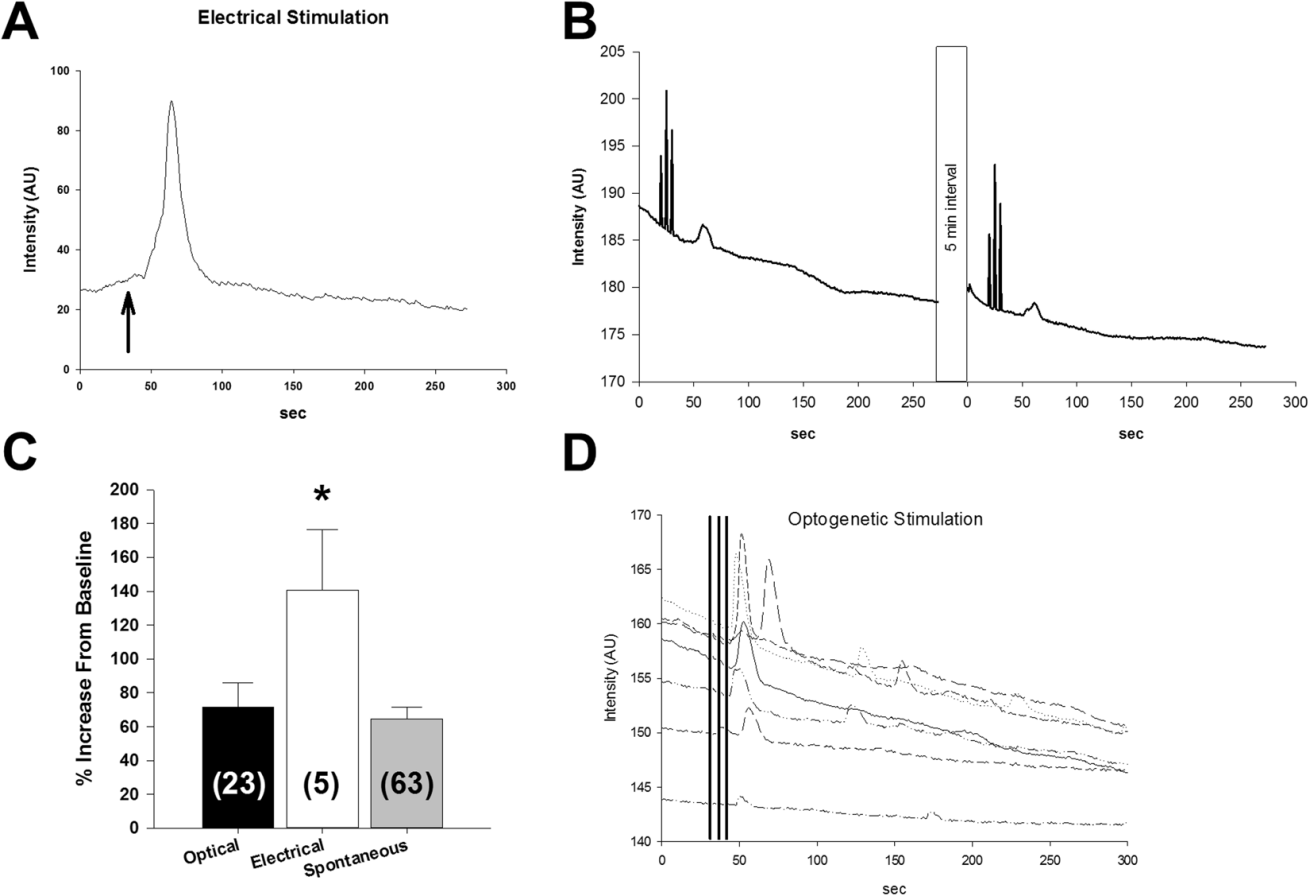
Sniffer cells respond to both electrically and optogenetically evoked ANG II release. (**A**) The SFO was electrically stimulated (arrow) and evoked release of AT1aR agonist that was detected in the MnPO. (**B**) The SFO was stimulated with 590 nm light and evoked release of AT1aR agonist that was detected in the MnPO with GCaMP + AT1aR sniffer cells. The light evoked sniffer cell response was reproducible. (**C**) Summary of GCaMP + AT1aR sniffer cell responses from *in vitro* slices. Optogenetic stimulation produced responses similar in magnitude to spontaneous events. Electrical stimulation produced responses that were greater than both light-evoked or spontaneous responses. (**D**) The SFO was stimulated with 470 nm light and evoked release of AT1aR agonist that was detected in the MnPO with R-GECO + AT1aR sniffer cells. *p 0.05.

Electrical stimulation-evoked increases in fluorescence in 5 GCaMP + AT1aR sniffer cells from 5 slices in 4 rats (Fig. 4A). There was a mean response latency of 15.8 ± 4.4 sec from the start of the electrical stimulation. There was a mean increase in peak fluorescence of 140.8 ± 35.7% over baseline fluorescence. Light stimulation-evoked increases in fluorescence in 23 GCaMP + AT1a sniffer cells from 5 slices in 4 rats (Fig. 4B). There was a mean response latency of 33.0 ± 3.9 sec from the start of the light stimulation. There was a mean increase in peak fluorescence of 71.4 ± 14.6% over baseline fluorescence. There was a significant effect of stimulation (F(3, 88) = 3.18, p < 0.05) on the amplitude of the sniffer cell responses. Electrical stimulation-evoked a significantly larger sniffer cell response when compared to the spontaneous events (p < 0.05, Figure 9). Light-evoked responses were not significantly different from spontaneous responses (p = 0.65, Fig. 4C).

Optogenetic stimulation-evoked increases fluorescence in 7 R-GECO + AT1aR sniffer cells on 2 slices from 1 rat. There was a mean response latency of 10.4 ± 1.1 sec from the start of the electrical stimulation. There was a mean increase in peak fluorescence of 2.7 ± 0.8% over baseline fluorescence (Fig. 4D). Light-evoked responses were not significantly different from spontaneous responses (p = 0.2).

## Discussion

In the above experiments, CHO cells stably transfected with AT1aR and either GCaMP or R-GECO plasmids yielded colonies that expressed their corresponding proteins. Select colonies from each condition became our workhorse sniffer cells. However, there was variability in the baseline expression of GCaMP and R-GECO fluorescence of these sniffer cells. This did not appear to affect the ability of the sniffer cells to selectively respond to ANG II. In control experiments with isolated AT1aR sniffer cells, exogenous application of ANG II produced a robust increase in fluorescence that was blocked by bath application of Losartan confirming that the cultured sniffer cells can detect ANG II and that ANG II initiates the increase in fluorescence through the activation of AT1aRs. Furthermore, bath application of other common neurotransmitters failed to increase cultured sniffer cell fluorescence indicating changes in sniffer cell fluorescence were specific to AT1aR activation.

The cultured sniffer cells demonstrated specific AT1aR activation; however, they did not exhibit convincing dose-response curves for either ANG II or ANG III. The dose-dependent responses were variable, particularly at higher doses. This variability in the dose response is likely due in part to the variability in baseline fluorescence of the cultured sniffer cells as well as variability in the expression of the AT1aR. Sniffer cells with low baseline fluorescence yielded poor signal-to-noise ratios, whereas high baseline fluorescence was associated with ceiling effects. The middle baseline fluorescence sniffer cells yielded the best responses and future sniffer cell production efforts will focus on standardizing expression within cell populations. The lower detection threshold in the dose response for ANG III (compared to ANG II) is consistent with reports showing a greater sensitivity of the AT1aR for ANG III over ANG II^29^. Angiotensin converting enzyme 1 (ACE1) is responsible for converting ANG I into ANG II as well as degrading bradykinin. We show here the cultured sniffer cells were not sensitive to bradykinin indicating that future studies using these sniffer cells to investigate ANG II signaling and manipulations of ACE1 will not be confounded by effects of bradykinin.

Suspended sniffer cells were then applied to the superficial surface of an *in vitro* brain slice. Sniffer cells adhered well and remained stationary on the slice despite continuous perfusion of aCSF in the recording chamber. Sniffer cells placed on the MnPO exhibited spontaneous increases in fluorescence. The spontaneous increases in fluorescence were inhibited by TTX suggesting that an action potential-dependent release mechanisms was involved. Furthermore, the spontaneous increases in fluorescence were completely blocked by bath application of Losartan. Spontaneous activity observed in the plated preparation was independent of AT1aR activation and likely characteristic of an unhealthy sniffer cell. Conversely, spontaneous activity observed in the brain slice preparation was dependent on AT1aR activation. This difference indicates that the spontaneous activity of the sniffer cells in the brain slice preparation was receptor meditated and likely due to ANG II or a related peptide released in the MnPO. Given the amount of time that the slices were incubated and perfused before the experiments began, sniffer cells that had non-receptor mediated activity could have been washed off. Together, these results suggest that the spontaneous changes in fluorescence that were observed in sniffer cells on the MnPO could be due to the synaptic release of angiotensin peptides that are stored in presynaptic vesicles.

Recent studies have suggested that ANG II can leak into the brain from the periphery, especially in disease states, and is not synthesized in the CNS. While there is dispute about the source of ANG II in the brain (locally synthesized vs taken up from the periphery) there is strong evidence that dysregulation of ANG II signaling in the brain can contribute to hypertension. Here, spontaneous sniffer cell responses in slices were observed as early as 1 h and as late as 5 h following removal of the brain from the rats. This persistent observation of spontaneous sniffer cell responses argues in favor of local synthesis of ANG II (or ANG III) within the brain as opposed to a peripheral reuptake mechanism as the source of ANG II signaling within the brain.

These data indicate that the sniffer cell responses are specific to AT1aR ligands and they are not sensitive to other compounds released by the neural tissue. However, while the current study shows that sniffer cells placed on a brain slice exhibit AT1aR activation, further studies are required to determine the contribution of various AT1aR agonists (e.g. ANG II vs ANG III) *in vitro*.

In addition to observing spontaneous responses, we were able to evoke sniffer cell responses by stimulating the pathway between the SFO and the MnPO. Electrical stimulation of the SFO efferent produced an increase in the fluorescence of sniffer cells placed on the MnPO. Optogenetic stimulation of the SFO was also effective in generating sniffer cell responses on the MnPO, although these responses were not as robust as that seen with electrical stimulation. However, optogenetics allow the activation of specific cell populations. This approach would be useful in future studies investigating the contribution of specific cell populations to ANG II signaling in the brain.

The results indicate that sniffer cells specific for ANG II and ANG III can be used to detect release of these peptides *in vitro* consistent with earlier studies focusing on vasopressin^23,24^ and oxytocin^25,26^. While these previous studies have typically used transient transfections, our studies have extended this approach to the creation of stable cell lines that might limit some of the variability of the expression of the receptor of interest and the calcium indicator. Additionally, the use of different genetically encoded Ca^2+^ sensors enhances the versatility of sniffer cells in conjunction with optogenetics or with other sniffer cells designed to detect different neuropeptides. Currently, it is difficult to establish a dose response with the sniffer cells which is due, in part, to the variability in expression of the Ca2+ sensors. While it is possible to select specific cells for analysis in the cell culture experiments to establish more reliable dose response curves, it is not feasible to do so in the *in vitro* slice preparation as sniffer cells will adhere randomly to the region of interest on the slice. Additionally, the apparent endogenous c-myc expression in the CHO cells and the lack of antibodies specific for the receptor makes quantification of AT1aR expression difficult. Future iterations of the sniffer cell line will include combining a fluorescent tag with AT1aR plasmid to allow fluorescent activated cell sorting based not only on expression of the Ca2+ indicator but also on AT1aR expression to reduce variability.

Our preliminary results support the existence of a brain renin-angiotensin system that features spontaneous and demonstrate SFO-evoked release of angiotensin peptides in the MnPO. These observations are consistent with a previously proposed pathway from the SFO to the MnPO that includes ANG II as a neurotransmitter^8,16,30^. Additional experiments will be required to explore further angiotensin synthesis and the mechanisms governing its release in the nervous system.

## Methods

### Solutions, reagents, and clones

The angiotensin type 1a receptor (AT1aR) plasmid (Agtr1a/pCMV6-Entry) (Agtr1a accession #: NM_030985) was obtained from Origene (Cat #RR215252), and the GCaMP plasmid (pGP-CMV-GCaMP6m from Douglas Kim) and R-GECO plasmid (CMV-NLS-R-GECO from Robert Campbell, University of Alberta, Canada) were obtained from Addgene (plasmid #40754 and #32462, respectively). R-GECO cDNA was then amplified by PCR with primers containing NotI (5′) and XbaI (3′) sites and subcloned into the p3XFLAG-CMV-7.1 vector (Sigma-Aldrich). However, this vector is not neomycin resistant, so the 3XFLAG-R-GECO cDNA was amplified by PCR with primers containing EcoRI (5′) and XbaI (3′) sites and subcloned into the pcDNA3.1+ vector (Thermo-Fisher), which is neomycin resistant. The resulting plasmids used to make stably transfected sniffer cells were neomycin resistant. Primer sequences used are shown as follows: (1) 5′-NOT-R-GECO-3′: 5′-ATAGCGGCCGCGGTCGACTCATCACGTCGTAAGTGGAATAAGG-3′; (2) 3′-XbaI-R-GECO-5′: 5′-GCTCTAGACTACTTCGCTGTCATCATTTGTACAAACTCTTCGTAGT-3′; (3) 5′-EcoRI-3XFLAG-R-GECO-3′: 5′-ACGCGAATTCGTTTAGTGAACCGTCAGAATTAACCATGGACTACAAA-3′. A Myc-DDK tag is located just C-terminal of Agtr1a cDNA. The antibodies used were monoclonal anti-Myc (Clone 9E10) (BioLegend), polyclonal anti-GFP (Invitrogen), monoclonal ANTI-FLAG^®^ M2-Peroxidase (HRP) antibody (Sigma Aldrich), and monoclonal anti-GAPDH (Millipore).

### Sniffer cells – Stable CHO cell lines

A kill curve experiment was conducted to determine the minimum antibiotic concentration used to select stably transfected CHO cells. CHO cells (ATCC, Manassas, VA) were exposed to different concentrations of G418 antibiotic that ranged from 0 to 1,600 μg/mL G418 (Roche, Sigma-Aldrich, St. Louis, MO), and, from this data, a concentration of 400 μg/mL G418 was chosen for the selection experiments. Normal CHO cell full media (F12K (ATCC), 10% FBS (Sigma-Aldrich), 1% penicillin-streptomycin (Corning)) was used for the first 2 days of transfection: *Day 0:* 90% confluent CHO cells in 35-mm dish were transfected with 250 ng/mL each of AT1aR and either GCaMP or R-GECO plasmids using Lipofectamine 3000 (Invitrogen, San Diego, CA); *Day 1:* Cells were passaged and plated onto 100-mm dishes so that they would be 10% to 20% confluent on Day 2; *Day 2:* 48 hours after transfection, media was replaced to full media with G418 (F12K, 10% FBS, 400 μg/mL G418). This G418 selection media was changed and refreshed every 3–4 days for the next 8–10 days. Then, the media was replaced with 5 mL of 1% low-melting agarose (Sigma-Aldrich, St. Louis, MO) in F12K for solid support. Twelve foci (colonies) were selected using cloning cylinders (Scienceware) and punched out by pipetting up-and-down 50 μL of 0.05% Trypsin-EDTA (Corning, Tewksbury, MA). Cells from each foci were seeded into one well in a 12-well plate and grown to 100% confluency (3–4 days). The cells were further expanded for Western blot analysis and functional assays. Stably transfected sniffer cell colonies were selected based on strength of AT1aR and either GCaMP or R-GECO protein expression levels.

### Cell Lysis and Western blot

Stably transfected cells were harvested and lysed using 600 µL of 1× RIPA buffer: 50 mM Tris-HCl, pH 8; 150 mM NaCl; 1% Triton X-100; 0.5% sodium deoxycholate; 0.1% SDS; 0.2 mM EDTA, protease inhibitor cocktail tablets. The cell extracts were sonicated, and insoluble material was spun down at 30,000 × g for 20 min. Ten µL from each sample was mixed with 5 µL of SDS-loading buffer dye and loaded onto 8% Tris-glycine SDS-PAGE gels. Gels were transferred onto PVDF membrane. Western blots were generated using the following primary antibodies: (1) Anti-GFP polyclonal at 1:1,000 dilution to detect GCaMP (Cat #A-11122 - Thermo Fisher Scientific, Walltham, MA) + secondary Goat Anti-Rabbit IgG (H + L) HRP-Conjugate at 1:5,000 dilution (Cat #1706515 - Bio-Rad, Hercules, CA); (2) Purified anti-c-Myc antibody (9E10) at 1:1,000 dilution (Cat #626801 - BioLegend, San Diego, CA) + secondary Goat Anti-Mouse IgG (H + L) HRP-Conjugate at 1:5,000 dilution, (Cat #1706516 - Bio-Rad, Hercules, CA); (3) Anti-GAPDH monoclonal at 1:2,000 dilution (Cat #MAB374 - Millipore-Sigma, Burlington, MA) + secondary Goat Anti-Mouse IgG (H + L) HRP-Conjugate at 1:5,000 dilution. Western blots for R-GECO sniffer cells used a monoclonal ANTI-FLAG^®^ M2-Peroxidase (HRP-conjugated) antibody at 1:1,000 dilution (Cat #A8592 - Sigma Aldrich) and anti-c-Myc antibody (9E10) at 1:1,000 dilution (sc-40 – Santa Cruz Biotechnology Inc., Dallas, TX). Blots were blocked using 5% dry milk in 1X PBS +0.1% Tween-20 (Sigma-Aldrich). All blots were incubated at room temperature. Incubation time periods for primary and secondary antibodies were 1 hour each, with in-between 3× washes using 5% milk in 1X PBS-Tween-20. Blots underwent 5× final wash after secondary-antibody incubation before application with enhanced chemiluminescence (ECL) substrate. All blot exposures were developed using X-ray film. Additional blots using alternate c-myc antibodies are described in the Supplemental Methods.

### Imaging

On the day before experiments, sniffer cells were plated onto coverslips coated with poly-L-lysine so that they would be 50% to 70% confluent on experiment day. These cells were washed of media 3× times with room temperature (25 °C) artificial cerebrospinal fluid (aCSF) containing (in mM): 126 NaCl, 3.0 KCl, 2.0 CaCl_2_, 2.0 MgSO_4_, 1.25 NaH_2_PO_4_, 26 NaHCO_3_, 10 and D-Glucose (300 mOsm, pH 7.4). Cover slips were then mounted on an open bath imaging chamber (RC-26, Warner Instruments, Hamden, CT) and continuously perfused with aCSF (25 °C, 1–2 ml/min). Drugs were bath applied for a period of 1 min then washed out with aCSF. Sniffer cells were imaged using epifluorescence on an Olympus IX81 inverted microscope. Changes in GCaMP fluorescence measured at 514 nm in response to 488 nm excitation (560/600 emission/excitation for R-GECO) at 1 second intervals using a CCD camera (C10600-10B, Hamamatsu Photonics K.K., Japan) and digitally acquired using MetaFluor (Molecular Devices, San Jose, CA). Responses to bath applied drugs were measured as the ratio of the peak response to baseline fluorescence. Additionally, responses were compared to the response to bath application of ionomycin (5 µM).

The following bath applied compounds were obtained from Sigma-Aldrich (St. Louis, MO): Glutamate (50 µM), GABA (50 µM), Angiotensin II (10–500 nM), Ionomycin (5 µM). Carbachol (50 µM), Angiotensin 1–7 (0.1–100 nM), Bradykinin (0.1–100 nM), and Losartan (10 µM) were purchased from Tocris (Minneapolis, MN) and Angiotensin III (0.1–100 nM) was purchased from Alomone Labs (Jerusalem, Israel). Doses of Glutamate, GABA, and Carbachol were selected to be in excess of the EC_50_ for the respective receptors to maximize the chance to elicit a response in the sniffer cells.

### Animals

Experiments were performed according to the National Institute of Health *Guide for the Care and Use of Laboratory Animals* (8^th^ edition) under a protocol approved by the University of North Texas Health Science Center Institutional Animal Care and Use Committee (protocol #: IACUC-2018-0012). These experiments used 6-week old (250–300 g) adult male Sprague-Dawley rats (Charles River Laboratory, Wilmington, MA). Animals were individually housed in temperature-controlled rooms with a 12-hour light dark cycle with the light phase lasting from 7am–7pm. Surgeries were performed using aseptic technique, and post-operative infection prevented by subcutaneous administration of procaine penicillin G (30,000 U). A non-steroidal anti-inflammatory drug, carprofen (Rimadyl, 2 mg tablet po), was given before and after surgery for pain management.

### Microinjections

The red shifted channel rhodopsin viral vector (AAV2-CamKII-C1V1(E122T/E162T)-TS-mCherry) or channel rhodopsin (AAV2-hSyn-ChR2(E123A)-eYFP-WPRE) used in these experiments were obtained from the UNC Vector Core as provided by the Deisseroth laboratory. The channel rhodopsin virus was injected undiluted at a titer of 1.1 × 10^12^ genomic particles/ml.

Rats were anaesthetized with 2% isoflurane and their scalps were shaved and disinfected with alcohol and iodine. Each rat was placed in a Kopf (Tujunga, CA) stereotaxic head frame. To ensure accurate injections, skulls were leveled between bregma and lambda^31^. The injection coordinates used for the SFO were 1.5 mm posterior, 0.0 mm lateral, and 5.5 mm ventral from bregma^32^. After drilling a burr hole at the site of injection, a 30-gauge steel injector was lowered to the SFO and 200–300 nL of AAV delivered at a rate of 200 nL/min. The injector was connected to a Hamilton 5 µL syringe (#84851 Hamilton Reno, NV) by calibrated polyethylene tubing that was used to determine the injection volume. The injector remained inserted for 5 minutes before being slowly withdrawn. Gel foam was packed in to the opening in the cranium and absorbable antibiotic sutures were used to close the incision site.

### Slice and sniffer cell preparation

Two to three weeks following microinjections, each rat was anesthetized with 2% isoflurane and decapitated. Sagittal slices (300 µm) containing the MnPO and the SFO were cut using a Microslicer DTK Zero 1 (Ted Pella, Inc., Redding, CA) in ice cold (0–1 °C), oxygenated (95% O_2_, 5% CO_2_) cutting solution consisting of (in mM): 3.0 KCl, 1.0 MgCl_2_-6H_2_O, 2.0 CaCl_2_, 2.0 MgSO_4_, 1.25 NaH_2_PO_4_, 26 NaHCO_3_, 10 D-Glucose, 206 Sucrose (300 mOsm, pH 7.4). Slices were incubated at room temperature in oxygenated (95% O_2_, 5% CO_2_) artificial cerebrospinal fluid (aCSF) containing (in mM): 126 NaCl, 3.0 KCl, 2.0 CaCl_2_, 2.0 MgSO_4_, 1.25 NaH_2_PO_4_, 26 NaHCO_3_, 10 and D-Glucose (300 mOsm, pH 7.4) for a minimum of 1 h prior to recording.

During this 1 h incubation period, 80% to 90% confluent sniffer cells on 60-mm or 100-mm plates were trypsinized, suspended in 1X PBS, and counted using a hemocytometer. Cells were then spun down and suspended in aCSF at a concentration of 1.3–2.5 × 10^6^ cells/mL.

Slices containing the MnPO and SFO were transferred to a submersion recording chamber and superfused with aCSF^33^. Slices were visualized using an upright microscope equipped for epifluorescence (BX50WI, Olympus) and differential interference contrast optics. Then, aCSF flow was temporarily interrupted and 300 µL of sniffer cell suspension was applied to the slice via pipette. Sniffer cells were allowed 3 min to settle before aCSF flow was resumed. In slices from uninjected rats, a concentric Pt/Ir bipolar stimulating electrode (#CBBRE75, FHC, Bowdoin, ME) was placed in the fiber path between the SFO and the MnPO. Slices were stimulated at 100 Hz for 1 sec (100 µA, 0.1 ms pulse duration). In slices from rats injected with channel rhosopsin, borosilicate glass micropipettes (1–3 µm tip) containing aCSF as the internal solution and a fiber optic cable in the tip of the micropipette were positioned at the MnPO. SFO terminals were activated using 590 nm (red shifted channel rhodopsin) or 470 nm (channel rhodopsin) light stimulation (20 Hz, 1 sec, 2 ms pulse, 5 mW). Fluorescent images of sniffer cells positioned over the MnPO were imaged using CellSens Dimensions software (Olympus). Images were captured at 1 sec intervals and measurements in fluorescent intensity were taken.

## Supplementary information

Supplement
