# Supplementary material for: Sniffer cells for the detection of neural Angiotensin II in vitro
Source: Sci Rep. 2019 Jun 19;9:8820. doi: 10.1038/s41598-019-45262-4 (PMC6584535; doi:10.1038/s41598-019-45262-4)
Supplement: Supplementary file 1 — Supplement [file 41598_2019_45262_MOESM1_ESM.docx]

**Supplementary Information**

**Sniffer cells for the detection of neural Angiotensin II in vitro**

George E. Farmer^1^, Anna Amune^2^, Martha E. Bachelor^1^, Phong Duong^1^, Joseph P. Yuan^1^, J. Thomas Cunningham^1^

^1^Department of Physiology and Anatomy, University of North Texas Health Science Center at Fort Worth, Fort Worth, TX 76107

^2^Texas A&M University, College Station, TX

Corresponding author: J. Thomas Cunningham, PhD
CBH-351
Department of Physiology and Anatomy
UNT Health Science Center at Fort Worth
3500 Camp Bowie Blvd
Fort Worth, TX 76107
Ph: (817) 735-5096
Tom.Cunningham@unthsc.edu

**Supplemental Methods:**

Western Blot: Following experimental transfections and subsequent plating, cells were then collected and homogenized in 150uL of RIPA lysis buffer supplemented with protease inhibitor cocktail (1:200), DTT (1uM) and EDTA (1mM). Protein concentration was determined using the Pierce BCA Protein Assay Kit per manufacturer’s instructions. Equal amounts of protein (10ug) were then separated in a Bio-Rad Any KD polyacrylamide gel at 25mA for approximately 1 hour and transferred onto a PVDF membrane at 50V in a 4oC fridge for 2-3 hours. Following transfer membrane blots were blocked using 5% non-fat milk in TBST for 30 minutes at room temperature. After blocking, membranes were incubated with constant agitation in primary antibodies (c-MYC 1:1000, Anti-FLAG 1:1000, GFP 1:1000, and GAPDH 1:10000) in 1% TBST non-fat milk either 2 hours at room temperature or overnight at 4oC. Membranes were then washed with 10% TBST 2 times for 10 minutes each before being incubated with secondary antibodies (Goat Anti-Rb HRP 1:1000 or Goat Anti-Ms HRP 1:10000) in 1% TBST non-fat milk for 30 minutes at room temperature. Afterwards, membranes were washed with 10% TBST 2 times for 10 minutes each. Visualization of bands was performed using SuperSignal West Femto Maximum Sensitivity Substrate and imaged for 30-90 seconds. Band intensity was then quantified by densitometry using the National Institutes of Health Image J program and normalized to GAPDH levels (n = 4).


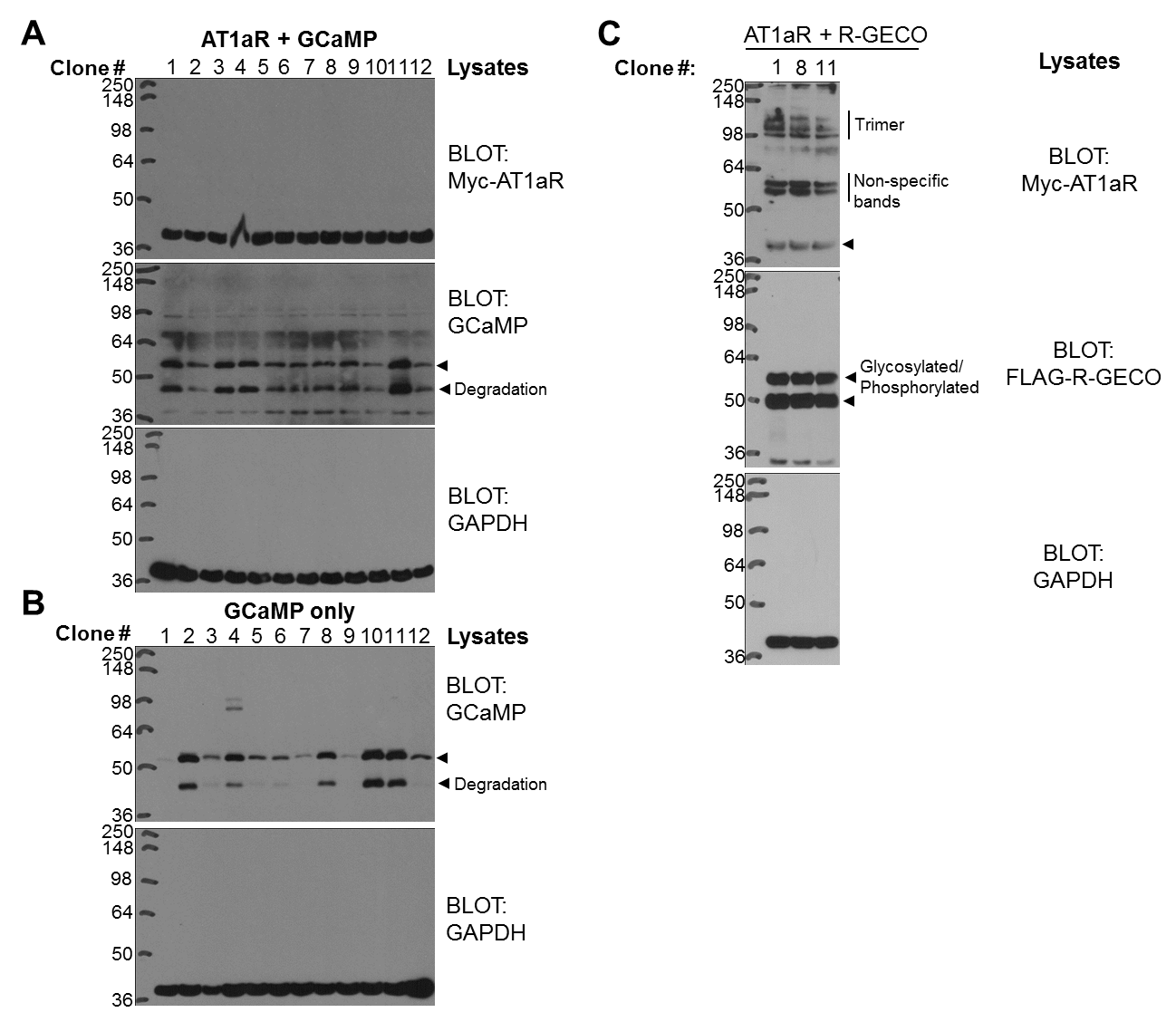
Supplemental Figure 1: Full Western blots showing selection of sniffer cells - stably transfected CHO cell colonies expressing high levels of A) GCaMP + AT1aR, B) GCaMP only, and C) R-GECO + AT1aR. Optimally expressed colonies selected: 1, 3, and 10 for GCaMP+AT1aR; 1, 10, and 11 for GCaMP only; and 8 and 11 for R-GECO+AT1aR.

Supplemental Figure 2: Western blots (A) show AT1aR expression, as inferred by the c-myc antibody, is present in all cell lines. (B) There was no difference in expression of the c-myc across cell lines. (C) There was more R-GECO expression in AT1a + R-GECO cells than R-GECO only cells. (D) There was more GCaMP expression in the GCaMP only cells compared to all other cells transfected with a Ca2+ indicator. * p < 0.05


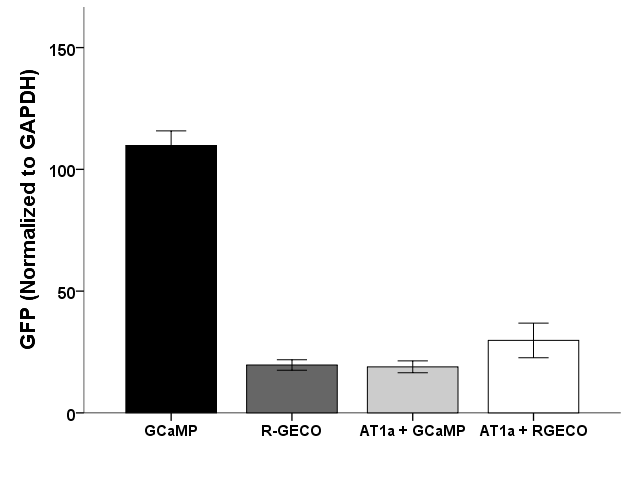


*****


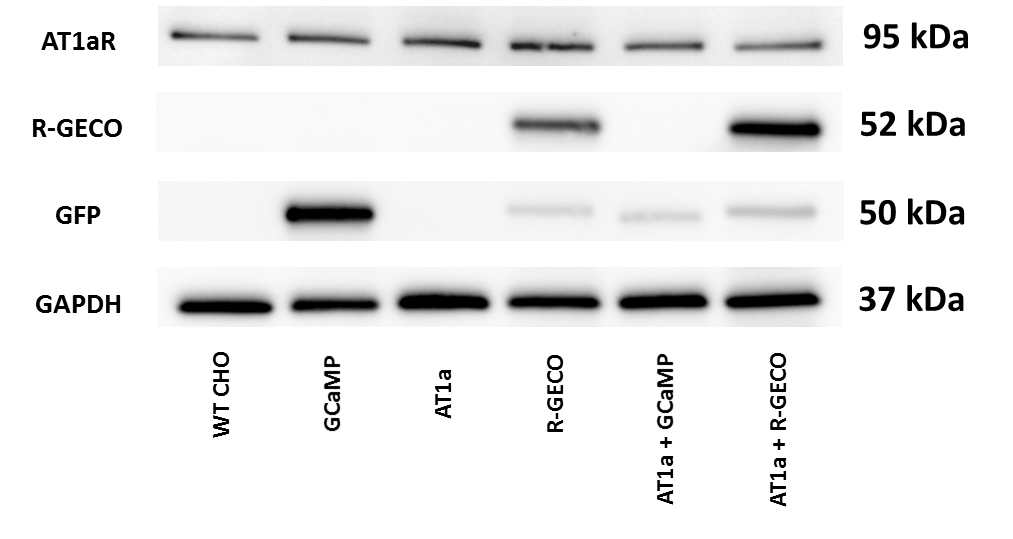

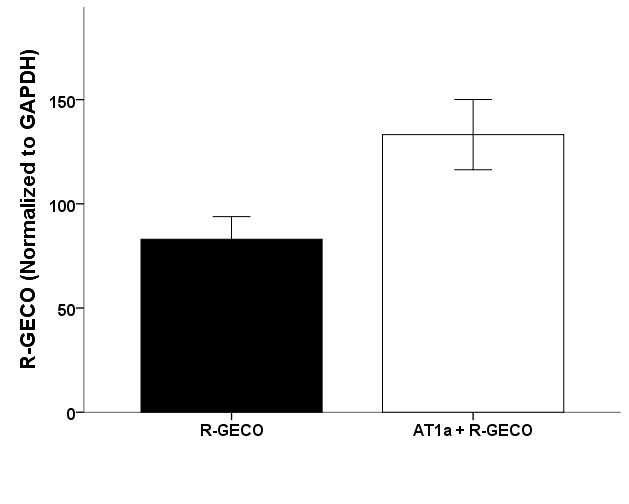


*****


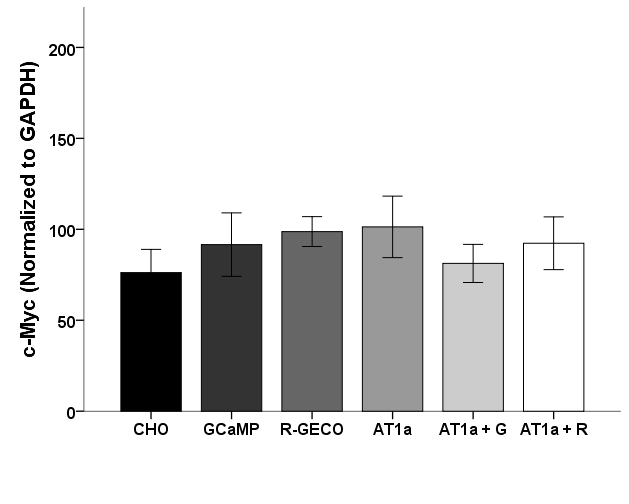


**A**

**B**

**C**

**D**


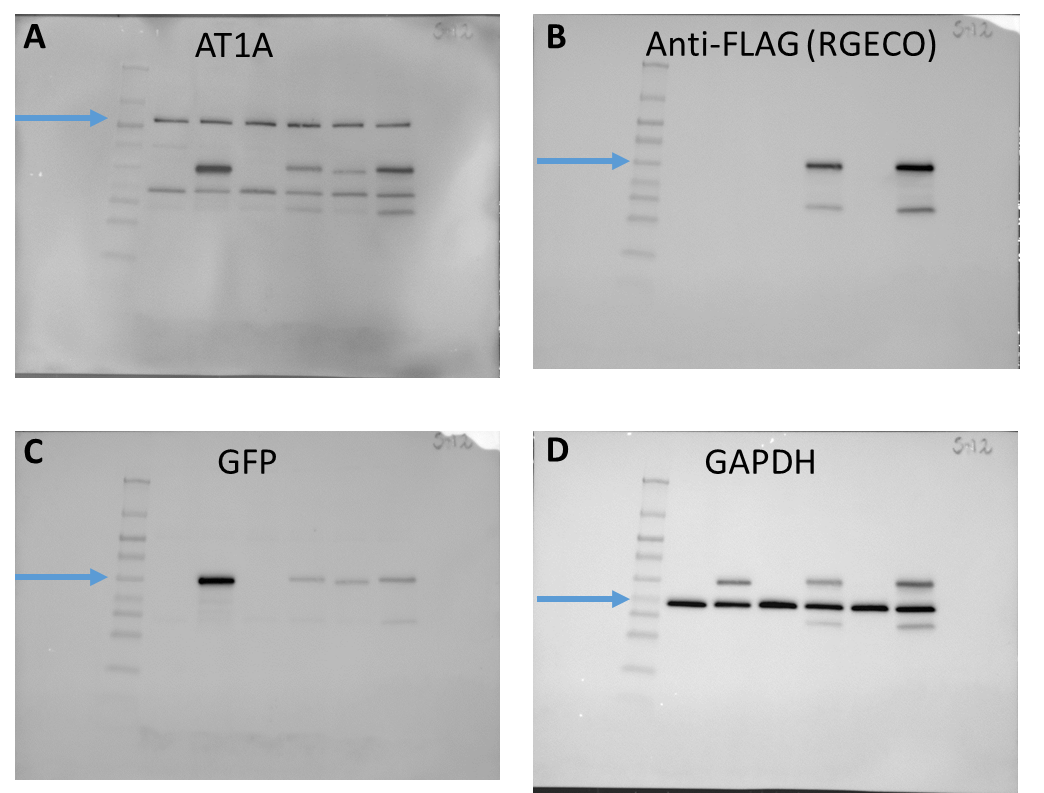
Supplemental Figure 3: Full Western Blots for (A) AT1aR, (B) R-GECO, (C) GCaMP, and (D) GAPDH depicted in supplemental figure 2A.

Supplemental Figure 4: Representative examples of a spontaneously active (black) and a healthy (gray) sniffer cell in response to bath application of Ang II (100 nM).
